# Ethics framework for treatment use of investigational drugs

**DOI:** 10.1186/s12910-020-00560-9

**Published:** 2020-11-18

**Authors:** Jan Borysowski, Andrzej Górski

**Affiliations:** 1 Department of Clinical Immunology, Medical University of Warsaw, Nowogrodzka Str. 59, 02-006 Warsaw, Poland; 2 Centre for Studies on Research Integrity, Institute of Law Studies, Polish Academy of Sciences, Nowy Świat 72, 00-330 Warsaw, Poland; 3 Laboratory of Bacteriophages, Ludwik Hirszfeld Institute of Immunology and Experimental Therapy, Polish Academy of Sciences, Rudolfa Weigla Str. 12, 53-114 Wrocław, Poland

**Keywords:** Expanded access, Compassionate use, Investigational drug, Right-to-try law, Declaration of Helsinki

## Abstract

**Background:**

Expanded access is the use of investigational drugs (IDs) outside of clinical trials. Generally it is performed in patients with serious and life-threatening diseases who cannot be treated satisfactorily with authorized drugs. Legal regulations of expanded access to IDs have been introduced among others in the USA, the European Union (EU), Canada and Australia. In addition, in the USA an alternative to expanded access is treatment under the Right-to-Try law. However, the treatment use of IDs is inherently associated with a number of ethically relevant problems.

**Main text:**

The objective of this article is to present a coherent framework made up of eight requirements which have to be met for any treatment use of an ID to be ethical. These include a justified need for the use of an ID, no threat to clinical development of the ID, adequate scientific evidence to support the treatment, patient’s benefit as the primary goal of the use of an ID, informed decision of a patient, fair access of patients to IDs, independent review, as well as the dissemination of treatment results.

**Conclusions:**

While this framework is essentially consistent with the legal regulations of expanded access of the USA, the EU, Canada and Australia, it is substantially wider in scope because it addresses some important issues that are not covered by the regulations. Overall, the framework that we developed minimizes the risks and threats, and maximizes potential benefits to each of the four key stakeholders involved in the treatment use of IDs including patients, doctors, drug manufacturers, and society at large.

## Background

Expanded access, also termed compassionate use, special access, early access or preapproval access, is the use of investigational drugs (IDs) outside of clinical trials [1]. The current increase in interest in expanded access results from a number of factors including the development of novel treatments for unmet medical needs, wide access of patients to information about new drugs in the Internet and high activity of patient advocacy groups [2]. In response to the needs of the growing number of patients many countries including the USA, most Member States of the European Union (EU), Canada, Australia, Japan and Brazil have introduced legal regulations to enable the use of IDs outside of clinical trials [3–5]. In general, expanded access treatment can be performed in patients with serious or life-threatening diseases who cannot be treated satisfactorily with approved drugs and are not eligible for enrollment in a clinical trial [3, 6]. Expanded access requests are reviewed by regulatory agencies; in addition, in some countries approval by an institutional review board (IRB) is required [3, 7]. Recently, in the USA the Right-to-Try Act was signed into law, thereby creating an alternative to expanded access regulations at the federal level [8]. This law allows patients with life-threatening diseases to request access to investigational treatments which have completed Phase 1 clinical trials without any oversight by the Food and Drug Administration (FDA). However, as is the case with expanded access, this pathway is dedicated for patients who exhausted all approved treatment options and are not eligible for participation in a clinical trial [8]. Note that the term ‘treatment use of IDs’ that will be used throughout this article refers to any use of those drugs outside of clinical trials including both expanded access and treatment under the Right-to-Try law.

While the treatment use of IDs is legally permissible in many countries, it is inherently associated with several ethically relevant problems. Generally, it involves four main stakeholders—patients, doctors, drug manufacturers, and society at large. Each of those parties has its interests and priorities which in some cases may be in conflict. For instance, too frequent use of IDs might pose a threat to the progress of clinical trials, which are essential for the introduction of new drugs to clinical practice [9]. Therefore, a key challenge is to find a reasonable balance between the needs of individual patients and those of the whole society [9, 10]. Individual patients in need can potentially benefit from the use of IDs, but are there any benefits to the society resulting from providing access to those drugs? Another important question is at what stage in its clinical development can an ID be used for treating patients? And how to ensure fair patient selection in programs involving the treatment use of IDs [2]?

Some of the important ethically relevant problems associated with the treatment use of IDs have not been addressed by the current regulatory systems [11–18]. For instance, none of these addresses the problem of fairness in selection of patients who seek access to IDs. Furthermore, there are no regulations pertaining to dissemination of results of programs providing access to IDs. Therefore, we developed a comprehensive ethics framework for the treatment use of IDs. This framework addresses the most important ethically relevant problems associated with the use of those drugs. It includes eight requirements which have to be met for the treatment use of IDs to be ethical. In this very specific context, we use the word ‘ethical’ to denote the treatment which maximizes the chance for benefits, and minimizes the threats and risks associated with treatment use of IDs to each of the four major stakeholders. We believe that these requirements are universal in that they are applicable to any use of IDs outside of clinical trials including both treatment of single patients and large expanded access programs.

## Main text

### Ethics framework for treatment use of investigational drugs

When designing our framework we considered the current legal regulations of expanded access of the USA [11], the EU [12–15], Canada [16], Australia [17, 18], and the Right-to-Try law [8]. Moreover, we refer to the Declaration of Helsinki (DH) by the World Medical Association (WMA). While the DH is generally a document containing principles pertaining to biomedical research involving humans, it also contains one paragraph (Par. 37) with guidance on the use of unproven treatments in clinical practice [19]. In addition, we searched scholarly literature on expanded access. Relevant articles were searched for in Medline through Pubmed. We used the keywords ‘expanded access’, ‘compassionate use’, ‘early access’, ‘managed access’, and ‘named patient’, and focused on articles published from 2009 through 2019. Based on the above sources, we identified the key ethically relevant problems associated with treatment use of IDs and developed our framework.

The framework is made up of eight main requirements. These include the following: (1) A justified need for the use of an ID; (2) No threat to clinical development of the ID; (3) Adequate scientific evidence to support the treatment; (4) Patient’s benefit as the primary goal of the treatment; (5) Informed decision of a patient; (6) Fair access of patients to IDs; (7) Independent review; as well as (8) The dissemination of treatment results. Each of these requirements is discussed along with its ethical relevance.

1. Justified need for the use of an investigational drug

Treatment with IDs with uncertain safety profile is associated with substantial risks to patients, often without a guarantee for cure. Moreover, widespread use of those drugs would be at odds with the fundamental principles of the current regulatory systems whose primary objective is to ensure that only safe and effective drugs are used in therapy [10]. It could also adversely affect the progress of clinical trials which are essential for the introduction of new drugs to clinical practice (see below, section No threat to clinical development of the ID; [9]). Overall, unrestricted use of IDs would pose threats not only to individual patients, but also to society at large. Therefore, treatment with IDs should be performed only in exceptional and well-justified cases.

In general, two main conditions have to be met to consider the treatment use of an ID. Firstly, those drugs should be used only in serious or life-threatening diseases [11, 13]. Exposing a patient to substantial risks associated with unproven treatments does not seem to be reasonably justified in diseases with a mild course and/or a good prognosis. Secondly, IDs should be used solely in situations when no authorized treatments can be satisfactorily used. If any authorized drug offers a reasonable chance for cure, then an unproven drug should not be used [1]. This results from a fact that authorized drugs which have passed through clinical trials are known to be reasonably safe and effective. Therefore if an authorized drug can be used in a given situation, it should be used in the first turn.

There are different situations in which no authorized treatment can be satisfactorily used. For instance, in some orphan diseases there is no authorized treatment at all [20]. In other cases, a doctor may consider treatment with IDs due to the development of resistance to authorized drugs. In fact, resistance is a great problem limiting the effectiveness of some drugs including antibiotics [21], antiviral agents [22] and anticancer drugs [23]. This is the likely reason why both infectious diseases and oncology belong to medical specialties with the highest number of requests for the use of IDs, at least in the USA [24]. Moreover, absolute contraindications to or the occurrence of serious side effects following the administration of authorized drugs can result in a necessity to use an unproven treatment. However, in each case both main conditions discussed in this section have to be met to deem the treatment use of an ID well-justified. Meeting those conditions prevents unnecessary exposure to unproven treatments of patients who can be treated with authorized drugs which have been shown to be reasonably safe and effective. Furthermore, it ensures a reasonable balance between the needs of individual patients and proper functioning of the drug regulatory systems.

Expanded access principles were originally developed with a view to enabling treatment use of IDs in a limited number of patients who meet the main criteria, especially have a serious or life-threatening disease that cannot be treated satisfactorily with approved drugs, and are not eligible for enrollment in a clinical trial. However, over recent years different initiatives have been undertaken to provide access to IDs to a larger number of patients [6]. These include, among others, the establishment of companies that facilitate access to IDs and the introduction of the Right-to-Try law. Nonetheless, for the reasons that we discuss in this article, we believe that the requirements discussed in this and the next section (No threat to clinical development of the investigational drug) should be preserved. Thus, growing awareness of doctors and patients regarding treatment use of IDs should not result in abandoning the main requirements for expanded access. Rather, it should lead to providing access to IDs to a larger number of patients who meet those requirements. However, a fact that a growing number of patients seek access to IDs, highlights a need for very careful evaluation of expanded access requests (see below, section ‘Independent review’).

2. No threat to clinical development of the investigational drug

Randomized controlled trials are the gold standard of studies to evaluate the safety and efficacy of IDs [25]. They are essential for the introduction of new drugs to clinical practice thereby providing benefits to the whole society. One of the greatest problems associated with the treatment use of IDs is that it may adversely affect the progress of trials [9]. If a large number of patients got an ID outside of a clinical trial, the enrollment in this trial could be limited; this could be of particular concern in rare diseases, where the number of patients is low. Patients may prefer the use of IDs outside of clinical trials for two main reasons. Firstly, in a clinical trial, the patient may be allocated to a control group and receive placebo or a standard of care rather than the ID. In fact, there are reports on actual clinical trials in which subjects received IDs in expanded access programs during their participation in the trials [26]. Secondly, enrollment in a clinical trial is associated with substantial burdens to subjects (e.g. performing tests essential to collect the data about the effects of the ID).

Overall the treatment use of IDs should be considered only in patients who for any reason cannot participate in clinical trials [11, 13]. This requirement is the primary safeguard to minimize potential threats to the progress of clinical trials. Clinical trial accrual faces many barriers and has already been inadequate [27]; failure to meet the requirement discussed in this section would have further aggravated this already very serious problem.

The current clinical trial enrollment criteria are in some cases quite restrictive. Consequently, only a small percentage of patients who exhausted all authorized treatment options may be eligible for participation in trials [28]. For all the remaining patients the treatment use of IDs is the only non-trial pathway providing them with access to new treatments. Apart from patients who do not meet enrollment criteria, the treatment use of IDs can be also considered in those who cannot participate in clinical trials for other reasons (e.g. a distant location of a trial center).

3. Adequate scientific evidence to support the treatment

To be ethical, the treatment use of IDs must be based on adequate scientific evidence. Otherwise, the patient is exposed to substantial risks without reasonable chance for cure. Based on the available data about the safety and efficacy of an ID as well as the patient’s condition, potential benefits and risks of the treatment can be estimated. Two key problems that need to be considered in this context include the likelihood and importance of potential benefits and whether the condition and prognosis of the patient justifies taking the risks associated with the treatment. However, one of the most difficult questions regarding the use of IDs is what level of evidence can be considered sufficient to start the treatment? In principle, firm conclusions about the safety and efficacy of drugs can be drawn following the completion of large Phase 3 trials; earlier phases yield only preliminary data about the effects of IDs [29]. Decisions regarding the treatment use of IDs are made on a case-by-case basis. Available evidence should be at first evaluated by a doctor, and subsequently verified by independent review (see below, section Independent review). One of the key factors in those considerations is the number of patients to be treated. Generally, a lower level of evidence may be deemed acceptable when treating single patients compared with large expanded access programs. Starting a large program based on inadequate evidence can result in harm to a greater number of patients. In line with this, a case was reported recently when treatment with an ID of one patient was approved by the FDA based merely on data from preclinical studies; the treatment was performed at a leading medical center and the results were published by a top-tier medical journal [30]. However, large programs involving hundreds or thousands of patients have been begun mostly after the completion of Phase 3 trials [31, 32].

The requirement presented in this section is important also because performing the treatment based on adequate evidence, along with informed consent of the patient (see below, section Informed decision of a patient) are the primary safeguards to shield the doctor in case of eventual litigation.

There are many studies reporting on successful use of IDs in different diseases [33–35]. On the other hand, the effectiveness of IDs found in other studies was modest [36, 37]. This is understandable due to a very large heterogeneity of expanded access studies. These involve different drugs, are performed in patients with a variety of diseases at different stages of advancement and with various comorbidities. Overall, in at least some patients, treatment with IDs can be of clinical benefit. This underscores the importance of the proper evaluation of requests for treatment use of IDs (see below, section Independent review).

With regard to the safety of IDs, according to the data collected by the FDA, serious adverse events resulting in a clinical hold on an investigational new drug (IND) development program occur extremely rarely in expanded access programs [24]. Thus, it appears that at least in the US, those programs are started at a stage when the data about the safety of IDs are sufficient.

4. Patient’s benefit as the primary goal of the treatment

Over the last years the sharp distinction between clinical care and biomedical research has become increasingly blurred [38, 39]. A perfect example of this problem is expanded access programs. On the one hand, as underscored by the guidance documents issued by both the FDA and the European Medicines Agency (EMA), those programs are not clinical trials and should have therapeutic purposes. Nonetheless, both agencies do allow safety and effectiveness data to be collected during the conduct of expanded access programs [13, 40]. Systematic collection of data is a feature of research rather than clinical practice. Thus, overall, expanded access programs constitute a unique combination of treatment and research aspects [7].

The primary objective of an expanded access program should always be the treatment of patients. When the data about the effects of IDs are collected in those programs, protocols should be designed as to maximize the direct benefits for actual patients; the collection of data should be a secondary objective. Moreover, in principle, expanded access programs are dedicated to patients ineligible for enrollment in clinical trials. Studies whose primary objectives include the evaluation of the safety and/or efficacy of IDs should not be classified as expanded access programs, but as clinical trials [41].

Concerns have been also expressed that some doctors could try to exploit treatment with IDs as a pretext of ‘pioneering’ novel therapies without paying sufficient attention to patients’ actual needs [10]. While the real scale of this problem is not known, cases of abuse associated with the conduct of experimental procedures in patients who have ran out of authorized treatments did occur even at some leading medical centres [42]. Furthermore, there is a risk that pharmaceutical companies might use expanded access programs as a way of promotion of new drugs among doctors, which could result in an increased demand for those drugs after their formal approval [43]. Such situations are of course ethically impermissible; in each case the treatment use of an ID must be performed primarily for the patient’s benefit.

5. Informed decision of a patient

The treatment use of IDs poses high risk of adverse events compared with most treatments employed in standard clinical care. Furthermore, patients with serious or life-threatening diseases who have exhausted all authorized treatments are considered particularly vulnerable in that they are very likely to overestimate potential benefits and underestimate risks associated with the use of new drugs which may give them the last chance for cure [44]. Therefore, particular care should be taken to ensure that the patient was conveyed an adequate range of information about the treatment and that he/she understood that information. Generally, to make an informed decision, the patient should be conveyed information concerning the nature and purpose of the treatment, its alternatives as well as potential benefits and risks. Two specific issues seem to be particularly relevant in the context of the treatment use of IDs. Firstly, the patient should be informed about the uncertain safety and effectiveness of the treatment. Secondly, he/she should be conveyed information about compensation for possible harms; if no compensation can be offered, the patient should be explicitly informed about this fact.

Some authors have rightly risen a problem of the capacity of vulnerable patients for making rational, informed decisions regarding treatment with IDs [44]. However, to resolve this concern, different methods can be employed in order to assess a patient’s capacity to appreciate his/her situation including potential consequences and to make rational choices [45]. Those methods seem to be particularly important in terminally-ill patients, a substantial percentage of whom may have their decision-making capacity impaired [46].

The main decision-making model that has been employed in the treatment use of IDs is informed consent. It is one of the main requirements for both expanded access and treatment under the Right-to-Try law in the USA [8, 11]. Informed consent is also required in Special Access Programs in Canada and Australia [16–18]. Moreover, it is listed in Par. 37 of the Declaration of Helsinki as one of the conditions of using unproven interventions in clinical practice [19]. While it is not explicitly required by Art. 83 (1) of Regulation (EC) No 726/2004 and Art. 1 of Directive 2004/27/EC of the European Parliament and of the Council [12, 15], it may be required by at least some of the national regulations which were not discussed in detail here.

We also believe that patients who have exhausted all authorized treatments may benefit from shared decision-making (SDM). SDM is a process involving a doctor and a patient which aims to reach a mutual treatment decision based on the best available medical evidence and a patient’s preferences and values. Over the last decade the importance of SDM has substantially increased and it is currently considered the cornerstone of patient-centered care [47]. SDM has been implemented in a variety of medical contexts including the treatment of patients with advanced cancer who consider participation in early phase clinical trials *vs* palliative care [48]. It appears that potential use of IDs outside of clinical trials, with palliative care as a major alternative, is also an example of preference-sensitive decision where the principles of SDM might be employed.

6. Fair access to investigational drugs

Treatment with IDs is not a part of standard medical care. The access to those drugs is very limited in view of applicable legal regulations and the production of only small quantities of IDs intended primarily for the needs of clinical trials. Of particular concern is a possibility that access to IDs might be easier for some individuals (e.g. those wealthier or well connected) at the cost of others. In order to prevent such abuses, every care should be taken to ensure fair access of patients to those drugs. In fact, ensuring fairness is considered one of the key ethical requirements of expanded access [49]. Overall, three main problems need to be taken into consideration in this context—the criteria for patient selection, the costs of the treatment and the availability of information about IDs.

While some pharmaceutical companies have provided patients with access to their IDs for a long time, there are no industry-wide best practices regarding patient selection. A reasonable practice is to select patients based on medical criteria considering the overriding principles of nonmaleficence and beneficence [2]. This means that higher allocation priority should be put on patients in whom no unacceptable harms are anticipated (nonmaleficence) and in whom scientific/medical evidence suggests a higher probability of benefit (beneficence). Social characteristics should not be considered apart from those that are strictly relevant to determining potential benefits of the treatment (e.g. the patient’s age). Insightful considerations were recently reported regarding how those principles were employed in a specific expanded access program [2]. However, detailed criteria for patient selection can vary by program.

The second important problem related to fairness in the access of patients to IDs is the cost of the treatment. Generally, the policies for charging patients for the treatment use of IDs vary by country. For instance, in the USA, in single patient expanded access, the sponsor can charge patients for the use of IDs, but can recover only the costs directly associated with making the drug available to the patient including the ID’s production cost [50]. However, in Canada, where manufacturers can charge for IDs too, there is no such requirement [16]. Detailed discussion of policies adopted in different countries is out of scope of this article. Overall, if the cost of the treatment is too high, fairness in patient access to IDs is substantially compromised. In fact, we believe that high costs of treatment can be one of the key factors limiting the access of patients to investigational treatments, especially in cases where relevant policies do not impose any limitations on the costs that manufacturers can demand. Furthermore, some insurance companies do not cover the costs of unapproved treatments. Thus in some cases high costs of those treatments may result is substantial financial harm to the patient; this is another important issue which should be taken into account in a discussion of ethics of expanded access [51, 52].

Another important issue is the availability of information about programs providing access to IDs outside of clinical trials. Unequal access to that information will limit fairness, as some patients may not be aware of the existence of novel treatments. A major step towards an increase in transparency of expanded access programs was the introduction in the USA of the 21st Century Cures Act, which mandated drug manufacturers to public posting of key data about their programs [53]. Of great help to patients can be also the Expanded Access Navigator developed by the independent not-for-profit Reagan-Udall Foundation. This navigator is the Internet-based resource intended to facilitate public access to information about possibilities of getting access to IDs outside of clinical trials. However, it contains information about single-patient expanded access only [54]. Furthermore, it was shown that a substantial percentage of patient advocacy organization websites do not post information about programs involving treatment use of IDs (although most of them do present information about clinical trials; [55]). This problem is important because it is the activity of those organizations that is considered a key factor in the development of expanded access [56]. Some inequities may be also associated with Internet-based clinical trial registers. While these were originally intended to make public information about clinical trials, some of them also post information about expanded access programs. For instance, as of 09/25/2020, 688 such programs were registered with ClinicalTrials.gov (CT.gov), the largest clinical trial registry in the world [57]. The Food and Drug Administration Amendments Act of 2007 (FDAAA) obliges sponsor of each applicable clinical trial being registered with CT.gov to specify whether the intervention evaluated in this trial is also available through an expanded access program [58]. If any such program is available, then it has to be registered with CT.gov. However, in view of a lack of relevant regulations or policies, other expanded access programs (that is, programs not associated with applicable clinical trials) likely have not been registered. Furthermore, other major registries, including the EU Clinical Trials Register (EUCTR) [59] and Australian New Zealand Clinical Trials Registry (ANZCTR) [60] do not have separate sections dedicated to expanded access. While expanded access programs can be registered with both EUCTR and ANZCTR, their number is very small compared with CT.gov [59, 60]. Thus, policies should be developed to provide the public with uniform information about different types of programs involving treatment use of IDs.

An important factor that can limit fairness in patients’ access to investigational treatments is that some doctors may be simply unaware of a possibility to use such treatments in their patients [61]. While this problem has not been investigated in detail, there are some data to indicate that (at least in some programs) most of expanded access requests come from very few doctors who treat patients with a given disease [2]. This problem could be addressed by training doctors regarding possibilities for use of investigational treatments.

7. Independent review

Each request for the use of an ID should be reviewed for two reasons: 1) such treatment is not a part of standard medical care and its potential benefits and risks may be hard to evaluate by a doctor [62]; 2) the use of an ID outside of a clinical trial may adversely affect clinical development of the drug [9]. Thus, the treatment use of an ID not meeting adequate standards would pose threats to both individual patients and society at large. As mentioned above (section Patient’s benefit as the primary goal of the treatment), different stakeholders involved in the treatment use of IDs, especially drug manufacturers and doctors, can have interests which may be not in accord with individual patients’ needs. Therefore, the review should be performed by a party independent from the drug manufacturer and the doctor who is to perform the treatment. It should focus on several key issues. First, all available data about the safety and efficacy of the ID should be evaluated in the context of the patient’s disease and prognosis; based on this, one can estimate whether the risks of the proposed treatment are acceptable and its expected benefits sufficiently backed by scientific evidence. Reviewers should also evaluate whether the treatment does not pose significant threat to clinical development of the ID. In addition, the qualifications of the doctor should be assessed. While this is not required by any regulations, in our opinion only doctors with relevant specialty should be permitted to use IDs. Our opinion results from two facts. First, those drugs are typically used in patients with serious or life-threatening diseases. Second, the available data about IDs are fragmentary and may be hard to assess by non-specialists. Therefore, in our view, the use of IDs requires specialty knowledge and doctor’s inadequate qualifications may unnecessarily expose the patient to additional risk.

The review of expanded access requests must be performed in accordance with applicable laws. In some countries, including the USA, Canada and Australia, expanded access is overseen by drug regulatory agencies [3]. In the EU, the EMA does not review compassionate use requests, which fall under jurisdiction of individual Member States [13]; detailed discussion of this issue is out of scope of this paper. In addition, in the USA, Italy and Australia, a requirement for expanded access is the review by an IRB [7]. However, no official guidelines have yet been developed regarding the review of expanded access requests and the relevant policies vary by IRB [63]. Moreover, independent expert committees like the recently established Compassionate Use Advisory Committee (CompAC) can play a role in the review of expanded access requests [2, 64]. CompAC is an international panel of experts with diverse backgrounds (medicine, bioethics, patient advocacy) that was established at an academic center to provide a major pharmaceutical company with recommendations about allocation of an ID in response to individual expanded access requests. Allocation criteria developed by CompAC with a view to a specific expanded access program can be adapted in future to other programs [2].

Unlike expanded access, treatment under the Right-to-try law does not require any institutional oversight [8]. However, we share the view of other authors [62] according to which the treatment use of IDs without the review by a regulatory agency is generally not advisable. We believe that independent review is essential for ensuring adequate level of public accountability of both expanded access and treatments performed under the Right-to-Try law.

8. Dissemination of treatment results

Both in the USA and the EU legal regulations were introduced to mandate making public the results of clinical trials [65, 66]. By contrast, the dissemination of the results of the treatment use of IDs is not required by law. Nonetheless, we believe that these should be disseminated. The use of IDs outside of clinical trials provides a unique opportunity to collect the data about the safety and effectiveness of those drugs in real-world settings. Those data very rarely replicate the results of randomized trials; their main advantage is that they complement the results of trials which are performed on patients meeting very stringent enrollment criteria [67]. For instance, large expanded access programs can be an important source of data on how investigational treatments work in real-world settings. Such programs are often multicenter, international, and can involve even thousands of patients [68, 69]. However, even treatment of single patients, where data collection is of less importance, can lead to important and novel findings which can be published as case studies [30, 70]. While uncontrolled studies are generally more prone to bias compared with randomized trials, their results may be further verified by clinical trials [70]. Generally, the importance of real-world data has significantly increased over recent years and some regulatory agencies including the FDA have been working on how to utilize those data in regulatory decision making [71]. However, a condition of making full use of those data is their publication. Making use of the generated data, along with the potential benefits to the treated patients, are the key factors to offset the risks and threats associated with the use of IDs. Those data, like data from clinical trials, can be of benefit to the whole society. The dissemination of the results of unproven treatments is promoted by Par. 37 of the Declaration of Helsinki [19].

## Conclusions

The treatment use of IDs has become a very complex enterprise involving an interplay of sometimes conflicting interests and priorities of different stakeholders, especially patients, doctors, drug manufacturers and society at large. It is also associated with a number of ethically relevant problems. IDs can be used both in single individuals and groups of patients. Particularly difficult to design and run are large, often international, expanded access programs which can involve thousands of patients [72]. However, we believe that in each case the treatment use of IDs has to meet the eight requirements that were discussed in this article. Meeting these conditions will reduce the risks and increase the likelihood of benefits to each of the four main parties involved in treatment use of IDs.

The framework is coherent in that all eight conditions have to be met for the treatment use of an ID to be ethical. However, the requirements presented in this article are not strict procedural guidelines or regulations. They are fairly general in nature and have to be adjusted to applicable legal regulations. For instance, in some countries, especially the USA, Australia, and Italy, expanded access requests have to be reviewed not only by a relevant regulatory agency, but also by an IRB; in other countries, there is no such requirement [7]. Another example is a possibility to start the treatment with an ID without the standard review and written permission of the FDA in emergency cases; however, in such cases the sponsor is obliged to submit to the FDA the necessary documentation within 15 days [40].

We are aware of a fact that our framework may not provide a simple and unequivocal answer to every clinical dilemma that can emerge in treatment use of IDs. For instance, in some cases potential benefits of different stakeholders involved in treatment use of IDs can be mutually incompatible. We believe that in such cases the fundamental principles of medical ethics should apply, especially the principle of nonmaleficence. The overriding value should always be the patient’s safety.

The considerations contained in this article may be helpful for those involved in the design, review and conduct of programs involving the treatment use of IDs. For instance, IRB members could refer to the considerations presented in this article when performing review of expanded access requests. Moreover, our framework can be used by doctors who consider investigational treatments in their patients.

## Data Availability

Not applicable.

## Abbreviations

CHMP: Committee for Medicinal Products for Human Use
CompAC: Compassionate Use Advisory Committee
EMA: European Medicines Agency
FDA: Food and Drug Administration
ID: Investigational drug
IRB: Institutional review board
SDM: Shared decision making
WMA: World Medical Association
